# The role of plasma cortisol in dementia, epilepsy, and multiple sclerosis: A Mendelian randomization study

**DOI:** 10.3389/fendo.2023.1107780

**Published:** 2023-03-15

**Authors:** Haiqi Li, Kaili Chen, Le Yang, Qiaoli Wang, Jiao Zhang, Jinting He

**Affiliations:** ^1^ Department of Neurology, China-Japan Union Hospital of Jilin University, Changchun, China; ^2^ Department of Endocrinology, Jilin Province People’s Hospital, Changchun, China

**Keywords:** Mendelian randomization, plasma cortisol, dementia, epilepsy, multiple sclerosis

## Abstract

**Background:**

Many clinical studies have shown a correlation between plasma cortisol and neurological disorders. This study explored the causal relationship between plasma cortisol and dementia, epilepsy and multiple sclerosis based on Mendelian randomization (MR) method.

**Methods:**

Data were taken from the summary statistics of a genome-wide association study, FinnGen consortium and United Kingdom Biobank. Dementia, epilepsy, and multiple sclerosis were used as outcomes, and genetic variants associated with plasma cortisol were used as instrumental variables. The main analysis was performed using the inverse variance weighted method, and the results were assessed according to the odds ratio (OR) and 95% confidence interval. Heterogeneity tests, pleiotropy tests, and leave-one-out method were conducted to evaluate the stability and accuracy of the results.

**Results:**

In two-sample MR analysis, the inverse variance weighted method showed that plasma cortisol was associated with Alzheimer’s disease (AD) [odds ratio (95% confidence interval) = 0.99 (0.98-1.00), *P* = 0.025], vascular dementia (VaD) [odds ratio (95% confidence interval) = 2.02 (1.00-4.05), *P* = 0.049)], Parkinson’s disease with dementia (PDD) [odds ratio (95% confidence interval) = 0.24 (0.07-0.82), *P* = 0.023] and epilepsy [odds ratio (95% confidence interval) = 2.00 (1.03-3.91), *P* = 0.042]. There were no statistically significant associations between plasma cortisol and dementia with Lewy bodies (DLB), frontotemporal dementia (FTD) and multiple sclerosis.

**Conclusion:**

This study demonstrates that plasma cortisol increase the incidence rates of epilepsy and VaD and decrease the incidence rates of AD and PDD. Monitoring plasma cortisol concentrations in clinical practice can help prevent diseases, such as AD, PDD, VaD and epilepsy.

## 1 Introduction

Dementia, epilepsy and multiple sclerosis (MS)are common neurological disorders for which there are reports of associations with endocrine or immune markers. Hormones are one of the major physiological regulators of brain development, and endocrine and immune system dysfunction may contribute to the development of dementia, epilepsy and MS (1–3).

Approximately 55 million individuals worldwide suffer from dementia, and that number is expected to rise to 78 million by 2030 and 152 million by 2050 (4, 5). In 2019, the number of people with dementia disability globally exceeded 25.27 million (6). Dementia ranks 7th among the top 10 causes of death worldwide, with over 1.62 million deaths from dementia (7). Dementia can be divided into degenerative and nondegenerative categories. The former involves Alzheimer’s disease (AD), dementia with Lewy bodies (DLB), Parkinson’s disease with dementia (PDD) and frontotemporal dementia (FTD), while the latter includes vascular dementia (VaD). AD accounts for 50%–70% of all dementia types (8), DLB is second only to AD in prevalence and accounts for 10%–15% of dementia (9), PDD accounts for approximately 3%-4% of dementia (10), FTD accounts for 5%–10% of dementia (11), and VaD is the most widespread type of dementia caused by a nondegenerative disease, accounting for 15%–20% of patients with dementia (12). Dementia and cognitive impairment are major global issues as the world’s population ages. Many clinical studies have shown that plasma cortisol has a direct relationship with cognitive impairment, and some observational studies have indicated a causal effect between plasma cortisol and dementia (13–15).

Epilepsy is a common neurological disorder worldwide and affects approximately 70 million individuals globally (16). Every year, there are 34 to 76 new cases diagnosed per 100,000 people (17). Epilepsy has high rates of disability and death, and the social and psychological burdens of the disease are severe, which seriously affects the quality of life of individuals with epilepsy (18). In recent years, many scholars have discovered that epilepsy has biological rhythms that are similar to the circadian rhythms of plasma cortisol (19). The circadian rhythms of plasma cortisol concentrations may affect the balance of neuronal excitability and inhibition, and a correlation exists between plasma cortisol and epilepsy susceptibility (20, 21).

Multiple sclerosis is an immune-mediated demyelinating disease of the central nervous system. It primarily affects individuals between the ages of 20 and 40 and is a major cause of disability in young adults, with serious social and economic burdens (22, 23). The Global Burden of Disease Study reports that the age-standardized MS prevalence is highest in high-income regions of North America, at 164.6 per 100,000 people, and is lowest in Asia (24, 25). Some studies have shown that hypothalamus-pituitary-adrenal (HPA) axis dysfunction is associated with the triggering of or increases in MS symptoms (26, 27).

Traditional observational research designs are case−control studies, whose findings are frequently influenced by confounding factors and where causal effects cannot be determined. In addition, randomized clinical trials can be limited by required ethical considerations. Therefore, valid strategies must be developed to identify causal relationships between exposure factors and response outcomes. Mendelian randomization (MR) is based on Mendel’s law of segregation, which states that allele pairs separate or segregate during gamete formation and randomly unite at fertilization (28). The single nucleotide polymorphisms (SNPs) of genes are determined at birth and are not altered by interference from external environmental and behavioral factors; thus, SNPs are considered instrumental variables (IVs), which can be used to enhance the exposure-outcome relationship, prevent reverse causality in the exposure-outcome association and avoid reverse causality. We implemented a two-sample MR analysis using summary statistics and attempted to explore the causal effect of plasma cortisol on dementia, epilepsy, and MS.

## 2 Methods

### 2.1 Study design and MR assumptions

**Figure 1:**
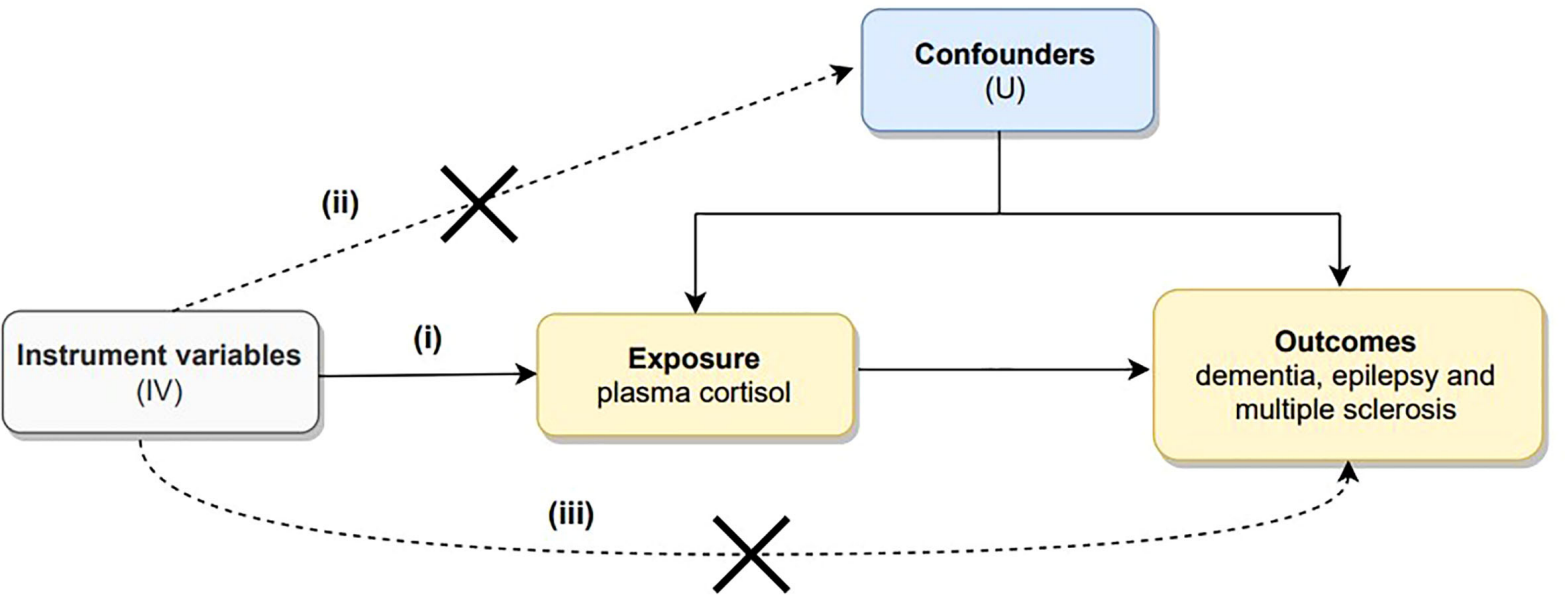
Directed acyclic graph illustrating the core assumptions of Mendelian randomization. There are three core assumptions: (i) there is a strong association hypothesis between genetic variants and exposure; (ii) genetic variants must be independent of confounding factors; and (iii) genetic variants can only impact outcome through exposure but not through other pathways.

MR is based on the following three core assumptions: (i) the association hypothesis: there is a strong association hypothesis between genetic variants and exposure; (ii) the independence hypothesis: genetic variants must be independent of confounding factors; and (iii) the exclusivity hypothesis: genetic variants can only impact the outcome through exposure and not through other pathways (29). The first hypothesis can be directly assessed by examining the strength of the association of genetic variants with risk factors; the second and third hypotheses can also be interpreted as not being influenced by pleiotropy and require sensitivity analyses for exclusion. Genetic pleiotropy means that genes affect more than one phenotypic trait (30). A directed acyclic graph of Mendelian randomization is depicted in **Figure 1**.

### 2.2 Instrumental variables

The exposure data were obtained from the CORtisol NETwork (CORNET), and included 12,597 participants (mean age 53.5 years, 59.2% female) from 11 Western European population-based cohorts (31). Participants’ blood was collected between 7:00 and 11:00 am, and plasma cortisol concentrations were determined by immunoassay. The z-scores of the plasma cortisol concentrations were log-transformed, and corrected for age, sex, and genetic control (31).

### 2.3 Outcome data

#### 2.3.1 Dementia

Dementia data were predominantly extracted from the United Kingdom Biobank (UKB) and FinnGen consortium. UKB was a large, detailed, population-based prospective study with over 500,000 participants aged 40–69 years conducted from 2006 to 2010 (32). The research involves medical records from a nationwide longitudinal follow-up and contains information on the phenotypes and genotypes of individuals. The UKB database was principally collected from surveys, sample testing, and genome-wide genotyping. The FinnGen consortium, which involved people over the age of 18 who resided in Finland, was a novel study that integrated genetic information with digital healthcare data (33). This database includes samples from hospitals, disease-based cohorts, and prospective epidemiological cohorts (34).

AD data were derived from the UKB, and a GWAS summary database published by Neale Laboratories in 2017. This database covered 26,757 patients with AD and 283,086 controls, all of whom were of European ancestry (35). In those data, AD was characterized as an anamnestic syndrome of the hippocampal type, with decreased memory, executive function, attention and word finding (36). SNPs associated with VaD were selected from the FinnGen consortium, which included 881 patients with VaD and 211,508 controls of European ancestry. PDD data were derived from the FinnGen consortium and included 267 cases of European ancestry and 216,628 normal subjects of European ancestry. For DLB, we used SNPs from the GWAS, including a sample of 2,591 cases and 4,027 healthy individuals (37). These participants were recruited from 44 institutions/consortia and diagnosed according to established consensus criteria, and all of the above participants were of European ancestry. SNPs associated with FTD were obtained from a GWAS dataset of 515 cases and 2509 controls of European ancestry. Because of the low population prevalence of FTD, the study population was obtained from 45 clinical centers in 11 countries (USA, Canada, UK, Netherlands, Belgium, Spain, Germany, Australia, Finland, France, and Sweden) and members of the International FTD Collaborative with the approval of the Institutional Review Board for cases (38). Detailed information is shown in **Table 1**.

**Table 1:** The characteristics of genome-wide association studies on outcome.

Outcome	Sample size for the outcome data		SNP	Consortium	Ethnicity
	Cases	Controls			
Alzheimer’s disease	26,757	283,086	10,894,596	UKB	European
Vascular dementia	881	211,508	16,380,457	FinnGen	European
Parkinson’s disease with dementia	267	216,628	16,380,459	FinnGen	European
Dementia with Lewy bodies	2,591	4,027	7,593,175	NA	European
Frontotemporal dementia	515	2,509	494,577	NA	European
Epilepsy	929	212,532	16,380,452	FinnGen	European
Multiple sclerosis	14,802	26,703	8,589,719	IMSGC	European

UKB, United Kingdom Biobank; IMSGC, International Multiple Sclerosis Genetics Consortium; SNP, single nucleotide polymorphism.

#### 2.3.2 Epilepsy

SNPs associated with epilepsy were gathered from the FinnGen consortium version R6, including a sample of 929 focal epilepsy cases and 212,532 normal controls of European ancestry. Diagnoses of epilepsy in the FinnGen consortium were based on G40 in the 10th edition of the International Statistical Classification of Diseases and Related Health Problems (ICD), and genotype data were obtained from the Finland Biobank and from digital health record data from the Finland Health Registry.

#### 2.3.3 Multiple sclerosis

We used summary-level data from the International Multiple Sclerosis Genetics Consortium (IMSGC), which included 14,802 patients with MS and 26,703 controls with European ancestry (39). Allele frequencies for SNPs in GWAS data of MS were obtained from 1000 genomic samples of European ancestry (40). Age, sex, and diagnosis criteria for the contributing cohorts were all accessible in the original GWAS.

### 2.4 Mendelian randomization analyses

We conducted a two-sample MR using the R programming language (Version 4.2.1; R Project for Statistical Computing, Vienna, Austria) (41). We obtained two samples of R programming packages from the MR analysis platform MR-Base.

We obtained the SNPs using the clump module in PLINK software (42). Because the number of SNPs with genome-wide significance is limited, we relaxed the association threshold with *P* < 5×10^−8^, a genetic distance of 10 000 kb and a linkage disequilibrium (LD) r^2^ < 0.3 to obtain the independent SNPs, which is consistent with the study of Larsson et al (43). The method was previously widely used in MR studies (44, 45). To remove confounding factors, we queried each SNPs on the website PhenoScanner (http://www.phenoscanner.medschl.cam.ac.uk/) and excluded SNP related to outcomes and confounders. To evaluate the strength of SNPs in this study F-statistics were calculated for individual SNPs, and F>10 indicated the absence of weak SNP bias. We extracted exposure SNPs from the outcome data and excluded those SNPs associated with outcomes (*P* < 5×10^−8^). Harmonization was then performed so that effect allele frequencies for exposure and outcomes correspond to the same effect allele.

To determine the causal effect between plasma cortisol and outcomes, we used inverse variance weighted (IVW) and MR−Egger regression, along with the weighted median, and maximum likelihood. Combining the IVW method with the Wald ratios (ratio of genetic association to outcome versus ratio of genetic association to exposure) of the causal effects of each SNP, where the odds ratio (OR) has a 95% confidence interval (CI) (46–48). The OR and 95% CI represented the risk for outcomes per standard deviation increase in circulating concentrations of plasma cortisol. Statistical significance was set at *P* < 0.05.

In MR analysis, we mainly used the IVW method to calculate the overall causal effects. To ensure the accuracy of the results, we further combined the inverse variance weighted freedom-effects (IVW-FE) method and the inverse variance weighted random-effects (IVW-RE) method to assess the causal effect between plasma cortisol and outcomes. The accuracy and reliability of the findings were evaluated by comparing the consistency of the findings across the different analytical methods.

We conducted a sensitivity analysis to alleviate the confounding impact of genetic pleiotropy on causal correlations. For the heterogeneity test, we used Cochran’s Q statistic to assess heterogeneity. If *P* > 0.05 for the result of the heterogeneity test, then there was no heterogeneity, and hypotheses (i) and (iii) in **Figure 1** were met, while the contrary suggested the existence of heterogeneity. For the pleiotropy test, pleiotropy test was assessed using the MR−Egger intercept method (49) to identify significant outliers in multiple SNPs. A *P*-value > 0.05 indicated that there was no horizontal pleiotropy. For the Leave-one-out sensitivity test, SNPs were removed one at a time, and the influence of each remaining SNP on the results was determined. If the results changed dramatically after eliminating a SNP, then SNP was considered an outlier and was removed from the analysis (50). Mendelian Randomization Pleiotropy RESidual Sum and Outlier (MR-PRESSO) regression analyses identified potentially pleiotropic outliers and estimated values after eliminating the outlier SNPs (51).

## 3 Results

### 3.1 Basic information on IVs

Summary statistics of instrumental SNPs as genetic IVs for plasma cortisol are presented in **Supplementary Table 1**. We selected three partially correlated SNPs (rs274952, rs12589136, and rs11621961) that are located in the genes *SERPINA6* and *SERPINA1*. These genes encode proteins that cleave the reactive center loop and release plasma cortisol from corticosteroid-binding globulin (45). Corresponding associations with dementia, epilepsy and MS were extracted. All selected IVs were strongly associated with dementia, epilepsy and MS (*P* < 5×10^-8^). IVs had an F-statistic > 10, suggesting that there were no weak effect IVs that would not introduce bias in the MR analysis.

### 3.2 Mendelian randomization

**Figure 2:**
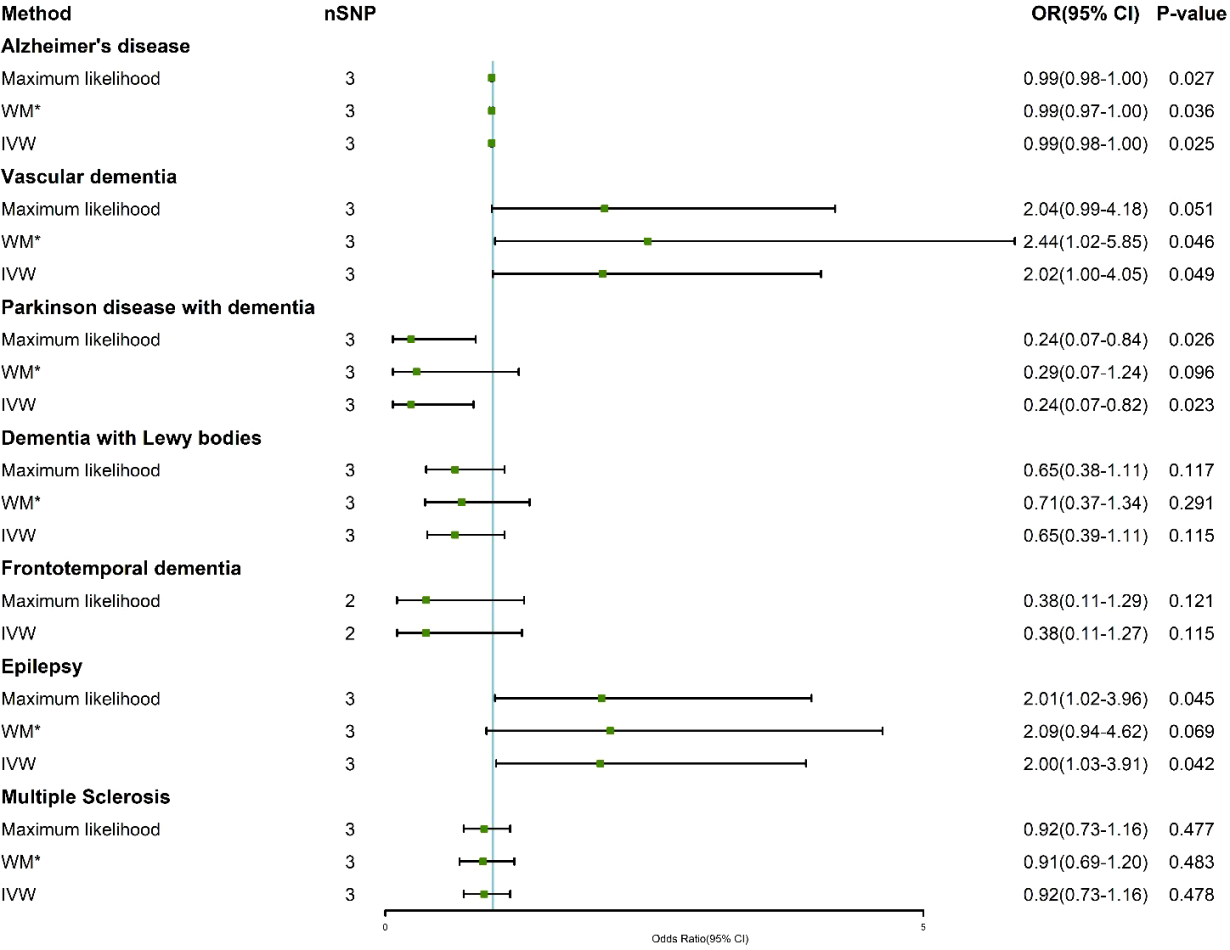
Association of genetically determined plasma cortisol and outcomes using the IVW, weighted median and maximum likelihood methods. IVW, inverse variance weighted; WM*, weighted median; OR, odds ratio; CI, confidence interval; SNP, single nucleotide polymorphism.

**Supplementary Tables 2–8** show the association of genetically predicted dementia, epilepsy, and MS with plasma cortisol using two-sample MR analysis. **Supplementary Figures 1–3** show scatter plots, forest plots, and funnel plots for MR analysis of the causal association between plasma cortisol and outcomes. According to the MR-IVW method, the causal effect between plasma cortisol and AD was statistically significant; the OR of AD per standard deviation decrease in plasma cortisol was 0.99 (95% CI 0.98-1.00), and the OR of VaD was 2.02 (95% CI 1.00-4.05) per standard deviation increase in plasma cortisol, with strength of evidence (*P* = 0.049). The results of MR are presented in **Figure 2**. Genetically predicted plasma cortisol was causally associated with PDD, and the OR of PDD per standard deviation decrease in plasma cortisol was 0.24 (95% CI 10.07-0.82, *P* = 0.023). A one standard deviation increase in genetically proxied plasma cortisol levels corresponded to a 2.00 normalized standard deviation-unit increase in epilepsy (95% CI =1.03-3.91, *P* = 0.042). There were no correlations between plasma cortisol and DLB, FTD and MS. The corresponding ORs and P-values were 0.65 (95% CI 0.39-1.11) *P* = 0.115 for DLB, 0.38 (95% CI 0.11-1.27) *P* = 0.115 for FTD and 0.92 (95% CI 0.73-1.16) *P* = 0.478 for PDD.

**Figure 3:**
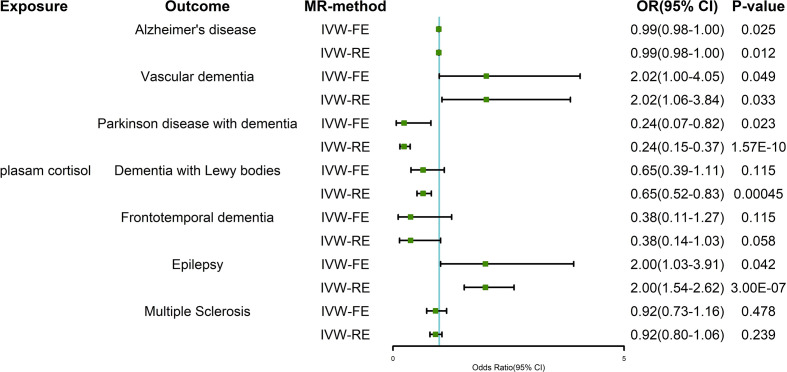
Two-sample Mendelian randomization estimates of the relationship between plasma cortisol and outcomes using IVW-FE and IVW-RE methods. IVW-FE, inverse variance weighted freedom-effects; IVW-RE, inverse variance weighted random-effects; OR, odds ratio; CI, confidence interval.

The results of the IVW-FE and IVW-RE methods were consistent, all showing that plasma cortisol was an independent risk factor for AD, VaD, PDD, and epilepsy. **Figure 3** shows the visualization results for the causal association between exposure and outcomes. We chose a forest plot to depict the results of the study. The corresponding ORs were 0.99 (95% CI 0.98-0.99) for AD, 2.02 (95% CI 1.06-3.84) for VaD and 0.24 (95% CI 0.15-0.37) for PDD using the IVW-RE method, with strength of evidence. A one standard deviation increase in genetically proxied plasma cortisol levels corresponded to a 2.00 normalized standard deviation-unit increase in epilepsy (95% CI =1.54-2.62, *P* < 0.001). The IVW-FE and IVW-RE methods showed similarities effect for AD, VaD, PDD, and epilepsy.

**Figure 4:**
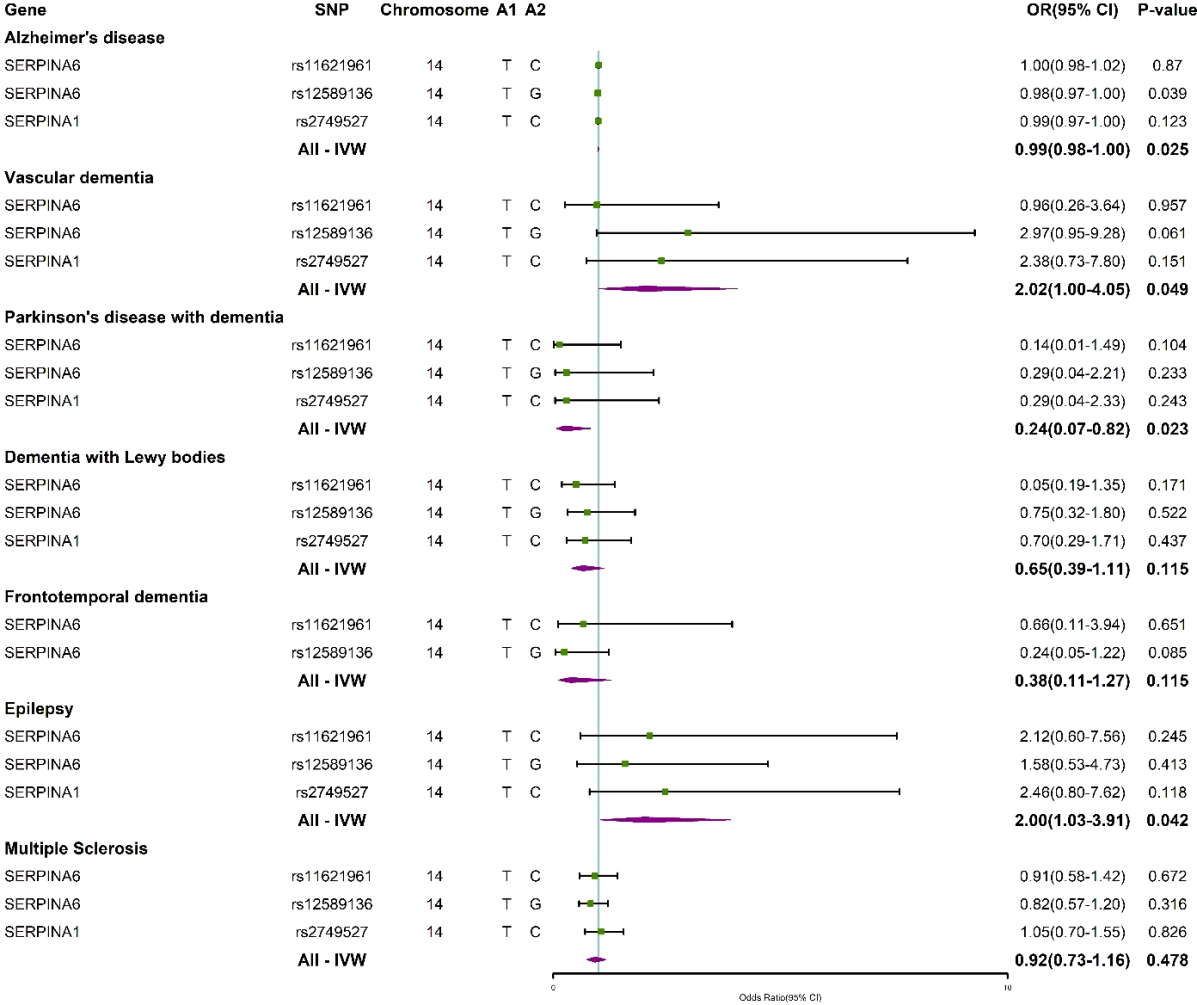
Forest plots summarizing the causal effects of specific plasma cortisol IVs and overall Mendelian randomization estimates associated with outcomes. IVW, inverse variance weighted; SNP, single nucleotide polymorphism; OR, odds ratio; CI, confidence interval.

Genetically predicted plasma cortisol was inversely associated with AD and PDD and positively related to VaD and epilepsy with the IVW method. The study further evaluated the connection between the three IVs and outcomes (**Figure 4**). The results showed that rs12589136 had a significant role in AD incidence with an OR of 0.98 (95% CI =0.97-1.00, *P* = 0.039).

### 3.3 Sensitivity analysis

In the MR analysis, we performed a heterogeneity test using both the IVW and MR−Egger methods. The results showed that there was no evidence of heterogeneity (*P* > 0.05) (**Supplementary Table 9**). To meet the third core assumption of the MR - the lack of horizontal pleiotropy, the MR−Egger intercept was used to assess the horizontal pleiotropy of IVs, and the results are presented in **Supplementary Table 10**, with *P* > 0.05 for the statistical results, suggesting no horizontal pleiotropy effect. In addition, we conducted “leave-one-out” sensitivity analysis. The black dots in each black line and each red line are on the same side of zero and therefore are not influenced by the removal of each IV, showing the robustness and reliability of the results of our MR analysis (**Supplementary Figure 4**). There were only three IVs in our study, therefore, MR-PRESSO regression analyses could not be performed. Sensitivity analyses using Cochran’s Q statistic, MR−Egger, and leave-one-out showed similar null findings, with no evidence of horizontal pleiotropy from the MR−Egger intercept.

## 4 Discussion

This two-sample MR study explored the causal relationship between plasma cortisol and dementia, epilepsy, and MS. The results of MR analysis demonstrated that genetic predisposition to higher plasma cortisol is associated with a decreased risk of AD and PDD. There was a positive correlation between plasma cortisol and VaD and epilepsy. Our findings do not suggest that plasma cortisol plays a major role in FTD, DLB, and MS. Neuroactive steroids have the ability to positively or negatively regulate the function of the nervous system (52). Plasma cortisol, one of the stress hormones affected by HPA, is involved in coordinating various processes throughout the body and brain, and is a stress response factor involved in the development of a variety of neuropsychiatric disorders. Many clinical studies have shown that cortisol is associated with anxiety and depression, cognitive impairment, and cerebrovascular disease (53, 54).

### 4.1 Plasma cortisol and dementia

Our results showed that higher genetically predicted plasma cortisol decreased the risk of AD and PDD, which is inconsistent with previous observational studies (55–59). Observational studies indicated that plasma cortisol levels were significantly higher in AD and PDD patients (58, 60). The outcomes of this investigation are different from previous findings. There are two possibilities for our results. First, there are studies demonstrating that genetically predicted plasma cortisol is associated with a reduced risk of obesity (61), and that plasma cortisol regulates energy metabolism by mobilizing glucose, fatty acids, and amino acids, which eventually reduces obesity (62–64). Obesity is also a risk factor for dementia (65). This indicates that increased cortisol concentrations reduce obesity, which somewhat decreases the incidence of dementia. Second, some limitations must be taken into account. Ferrari, E. et al. (66) showed that cortisol levels were significantly higher during the nighttime in elderly subjects. The GWAS database for plasma cortisol was restricted to samples collected in the morning, which had an impact on the MR analysis. In addition, there was nonrandom selection in the cohort, there were low response rates, and the individuals had a relatively high average education and general health status, which may have affected the results, particularly in the UK Biobank data.

This study demonstrates that higher plasma cortisol is associated with a higher risk of VaD (67). Reports of observational studies on cortisol levels in patients with vascular dementia are disputed, with some studies finding low levels of cortisol following cerebral infarction and others suggesting elevated cortisol concentrations (68, 69). Our investigation offered an important additional source for the evidence that plasma cortisol increased the risk of VaD. The stress response of the body and other neurohumoral factors can also affect the function of the HPA axis (70). Alterations in the HPA axis that result in higher plasma cortisol levels have been linked to corticoid-dependent hippocampal damage and poststroke brain disorders, which can cause cognitive impairment (71). The hippocampus and frontal cortex, which are critical for memory and learning, might suffer damage from elevated plasma cortisol levels (72). This interferes with the development of memory and learning, thus increasing memory loss and cognitive decline. As cerebral infarction proceeds, cerebral edema gradually increases, affecting the blood supply to the pituitary gland and inhibiting the function of the HPA axis, resulting in a decrease in adrenocorticotropic hormone and cortisol secretion (73).

Genetically predicted plasma cortisol was not associated with DLB or FTD, which is consistent with previous observational studies (74, 75). Although Woolley et al. (74) observed significantly lower plasma cortisol levels in patients with FTD, their findings suggested that the reduction in plasma cortisol is probably because of the production of ghrelin in the gastric mucosa after eating (76); thus, there is no clear causal association with FTD, which is consistent with the results of our study. Some researchers have shown that the anticholinergic activity of DLB occurs through reduced plasma cortisol levels, which are caused by severe autonomic parasympathetic dysfunction in DLB (75). Therefore, a bidirectional MR analysis can be undertaken in the future for plasma cortisol and DLB, and the causal association can be further defined.

### 4.2 Plasma cortisol and epilepsy

Using two-sample MR analysis, genetic variants were causally related to the risk of epilepsy, which is consistent with previous findings (77, 78). Epilepsy is a chronic neurological condition defined by spontaneous recurrent epilepsy (continuous synchronous discharge of neurons). The supraoptic nucleus of the hypothalamus is related to the HPA axis, which directly instructs the rhythm of plasma cortisol secretion. Circadian rhythms of plasma cortisol concentrations may alter several essential homeostatic processes in the body, including the balance between neuronal excitability and inhibition (79). Many researchers believe that plasma cortisol may modify neuronal excitability and epilepsy through GABA levels, which may have a substantial impact on signal transduction and epilepsy susceptibility. Some individuals are more susceptible to having epilepsy, especially when feeling stressed or anxious. Chronic stress decreases the synthesis of GABAergic neurotransmitters, and the loss may impair GABAergic inhibition and generate a neuronal state that is prone to hyperexcitability (21).

### 4.3 Plasma cortisol and multiple sclerosis

The present study did not find a causal association between plasma cortisol and the risk of developing MS, which is different from many observational studies (80, 81). MS is associated with a variety of neurological symptoms (82), including sensory disturbances, visual problems, fatigue, altered balance, cognitive dysfunction, anxiety and depression (83–88). Many studies have shown that the HPA axis plays an important role in controlling the disease process in MS (89, 90), and researchers have also revealed that anxiety and depression are significantly linked to higher levels of cortisol, especially in patients with relapsing-remitting MS (91, 92). The previous findings differ from the results of our study. This may be because the GWAS data of MS we used did not contain information on MS with depression or anxiety, so we could not clarify whether cortisol is causally associated with depression or anxiety symptoms. In addition, there is a significant sex difference in the incidence of MS, particularly in young women. This study did not classify MS patients by sex, and the causal correlation between sex differences and dysfunction of the HPA could not be clarified. Further research can be conducted l in the future.

This MR study should be confirmed or refuted when larger GWAS databases emerge for plasma cortisol. Nevertheless, our findings may trigger a discussion about the relevance and importance of the endocrine system, especially the HPA axis to the central nervous system.

### 4.4 Strengths and limitations

This study has several strengths. This was the first study to examine the causal association between plasma cortisol and dementia, epilepsy, and MS using data from GWAS. Compared to observational research, MR analysis minimized the effects of confounding factors. This study also has certain limitations. First, the GWAS data used in our study are limited in number and ancestry, and the relevant SNPs may not have been fully identified. The low sample size of this study may also affect the stability of the results. Second, this study investigated European ancestry for the exposure and outcome, so our results may not be applicable to other populations.

## 5 Conclusions

This study demonstrated that plasma cortisol increased the incidence rates of VaD and epilepsy and decreased the incidence rates of AD and PDD. Our MR results did not reveal evidence of a causal relationship between plasma cortisol and DLB, FTD or MS. Monitoring plasma cortisol concentrations can help prevent diseases in clinical practice.

## Data availability statement

Publicly available datasets were analyzed in this study. This data can be found here: https://gwas.mrcieu.ac.uk/.

## Author contributions

HL drafted the manuscript; KC and LY assisted in the preparation of the manuscript and performed the analysis; QW performed the analysis; JZ contributed to the data extraction; and JH designed the study. All authors contributed to the article and approved the submitted version.
