# Supplementary material for: The role of plasma cortisol in dementia, epilepsy, and multiple sclerosis: A Mendelian randomization study
Source: Front Endocrinol (Lausanne). 2023 Mar 15;14:1107780. doi: 10.3389/fendo.2023.1107780 (PMC10050717; doi:10.3389/fendo.2023.1107780)
Supplement: Supplementary file 1 [file DataSheet_1.docx]

Supplementary Material

The role of plasma cortisol in dementia, epilepsy, and multiple sclerosis: A Mendelian randomization study

**Haiqi Li^1†^, Kaili Chen^1†^, Le Yang^2†^, Qiaoli Wang^1^, Jiao Zhang^1^, Jinting He^1*^**

†These authors contributed equally to this work and share the first authorship

*** Correspondence:** Corresponding Author: Jinting He, e-mail: hejt@jlu.edu.cn

# Supplementary Tables

**Supplementary Table 1. Association between plasma cortisol instruments and outcomes in summary-level analyses**

| **SNP** | | | **Chr** | | **Position** | | | | **A1** | **A2** | | | **Exposure: Plasma cortisol** | | | | | | | | | | | **Outcome: Alzheimer’s disease** | | | | | |
| --- | --- | --- | --- | --- | --- | --- | --- | --- | --- | --- | --- | --- | --- | --- | --- | --- | --- | --- | --- | --- | --- | --- | --- | --- | --- | --- | --- | --- | --- |
|  |  |  |  |  |  |  |  |  |  |  |  |  | **Beta** | | | **SE** | | **P**-**value** | | | **F-statistic** | | | **Beta** | | | **SE** | | **P**-**value** |
| rs11621961 | | | 14 | | 94769476 | | | | T | C | | | -0.07659 | | | 0.013935 | | 3.97E-08 | | | 30.21173 | | | 0.0001188 | | | 0.0007245 | | 0.869765 |
| rs12589136 | | | 14 | | 94793686 | | | | T | G | | | 0.103402 | | | 0.014838 | | 3.32E-12 | | | 48.56318 | | | -0.001784 | | | 0.0008635 | | 0.0388097 |
| rs2749527 | | | 14 | | 94827068 | | | | T | C | | | -0.08139 | | | 0.012387 | | 5.21E-11 | | | 43.16746 | | | 0.0010855 | | | 0.0007029 | | 0.122508 |
| **Outcome: Vascular dementia** | | | | | | | **Outcome: Parkinson’s disease with dementia** | | | | | | | | | | | | **Outcome: Dementia with Lewy bodies** | | | | | | | | |  |  |
| **Beta** | **SE** | | | **P**-**value** | | | **Beta** | | | | | **SE** | | | **P**-**value** | | | | **Beta** | | | **SE** | | | | **P**-**value** | |  |  |
| 0.0028 | 0.0519 | | | 0.9576 | | | 0.1494 | | | | | 0.0918 | | | 0.1037 | | | | 0.0528865 | | | 0.0386301 | | | | 0.171005 | |  |  |
| 0.1126 | 0.0601 | | | 0.0607393 | | | -0.1276 | | | | | 0.1069 | | | 0.2325 | | | | -0.0293042 | | | 0.0458147 | | | | 0.522423 | |  |  |
| -0.0707 | 0.0492 | | | 0.1505 | | | 0.1016 | | | | | 0.087 | | | 0.243 | | | | 0.0288109 | | | 0.0370463 | | | | 0.436688 | |  |  |
| **Outcome: Frontotemporal dementia** | | | | | | | | **Outcome: Epilepsy** | | | | | | | | | **Outcome: Multiple Sclerosis** | | | | | | | |  |  |  |  |  |
| **Beta** | | **SE** | | | | **P**-**value** | | **Beta** | | | **SE** | | | **P**-**value** | | | **Beta** | | | **SE** | | | **P**-**value** | |  |  |  |  |  |
| 0.0314987 | | 0.0696539 | | | | 0.6511 | | -0.0577 | | | 0.0496 | | | 0.2449 | | | 0.0073268 | | | 0.01731 | | | 0.6721 | |  |  |  |  |  |
| -0.148616 | | 0.0862939 | | | | 0.08501 | | 0.0473 | | | 0.0578 | | | 0.4136 | | | -0.019901 | | | 0.0198509 | | | 0.3161 | |  |  |  |  |  |
| / | | / | | | | / | | -0.0734 | | | 0.0469 | | | 0.1177 | | | -0.003606 | | | 0.0163768 | | | 0.8257 | |  |  |  |  |  |

**Supplementary Table 2. Two-Sample MR Estimates of Relationship Between Plasma cortisol and Alzheimer’s disease**

| **Exposure: Plasma cortisol Outcome: Alzheimer’s disease** | | | | | | |
| --- | --- | --- | --- | --- | --- | --- |
| **Method** | **Beta** | **SE** | **P**-**value** | **or** | **or_lci95** | **or_uci95** |
| MR Egger | -0.053593 | 0.040326 | 0.410662 | 0.947818 | 0.875787 | 1.025773 |
| Maximum likelihood | -0.011582 | 0.005235 | 0.026947 | 0.988485 | 0.978395 | 0.998680 |
| WM* | -0.013642 | 0.006455 | 0.034555 | 0.986450 | 0.974049 | 0.999009 |
| IVW | -0.011396 | 0.005069 | 0.024554 | 0.988669 | 0.978896 | 0.998540 |

IVW, Inverse variance weighted; WM*, Weighted median

**Supplementary Table 3. Two-Sample MR Estimates of Relationship Between Plasma cortisol and Vascular dementia**

| **Exposure: Plasma cortisol Outcome: Vascular dementia** | | | | | | |
| --- | --- | --- | --- | --- | --- | --- |
| **Method** | **Beta** | **SE** | **P**-**value** | **or** | **or_lci95** | **or_uci95** |
| MR Egger | 3.594770 | 2.826551 | 0.424198 | 36.407327 | 0.142948 | 9272.524584 |
| Maximum likelihood | 0.712434 | 0.365858 | 0.051499 | 2.038948 | 0.995364 | 4.176672 |
| WM* | 0.892605 | 0.441011 | 0.042970 | 2.441481 | 1.028626 | 5.794946 |
| IVW | 0.701115 | 0.356363 | 0.049135 | 2.015999 | 1.002647 | 4.053519 |

IVW, Inverse variance weighted; WM*, Weighted median

**Supplementary Table 4. Two-Sample MR Estimates of Relationship Between Plasma cortisol and Parkinson’s disease with dementia**

| **Exposure: Plasma cortisol Outcome: Parkinson’s disease with dementia** | | | | | | |
| --- | --- | --- | --- | --- | --- | --- |
| **Method** | **Beta** | **SE** | **P**-**value** | **or** | **or_lci95** | **or_uci95** |
| MR Egger | 0.226460 | 5.019473 | 0.971298 | 1.254152 | 0.000067 | 23496.982177 |
| Maximum likelihood | -1.440195 | 0.644863 | 0.025527 | 0.236882 | 0.066929 | 0.838396 |
| WM* | -1.246188 | 0.760394 | 0.101240 | 0.287599 | 0.064793 | 1.276578 |
| IVW | -1.438013 | 0.631589 | 0.022797 | 0.237399 | 0.068843 | 0.818651 |

IVW, Inverse variance weighted; WM*, Weighted median

**Supplementary Table 5. Two-Sample MR Estimates of Relationship Between Plasma cortisol and Dementia with Lewy bodies**

| **Exposure: Plasma cortisol Outcome: Dementia with Lewy bodies** | | | | | | |
| --- | --- | --- | --- | --- | --- | --- |
| **Method** | **Beta** | **SE** | **P**-**value** | **or** | **or_lci95** | **or_uci95** |
| MR Egger | 0.622080 | 2.141414 | 0.820016 | 1.862798 | 0.028013 | 123.872417 |
| Maximum likelihood | -0.424721 | 0.271537 | 0.117785 | 0.653952 | 0.384069 | 1.113481 |
| WM* | -0.344294 | 0.310655 | 0.267739 | 0.708720 | 0.385514 | 1.302897 |
| IVW | -0.423540 | 0.268692 | 0.114956 | 0.654725 | 0.386673 | 1.108598 |

IVW, Inverse variance weighted; WM*, Weighted median

**Supplementary Table 6. Two-Sample MR Estimates of Relationship Between Plasma cortisol and Frontotemporal dementia**

| **Exposure: Plasma cortisol Outcome: Frontotemporal dementia** | | | | | | |
| --- | --- | --- | --- | --- | --- | --- |
| **Method** | **Beta** | **SE** | **P**-**value** | **or** | **or_lci95** | **or_uci95** |
| Maximum likelihood | -0.979417 | 0.630817 | 0.120515 | 0.375530 | 0.109064 | 1.293025 |
| IVW | -0.968205 | 0.614874 | 0.115339 | 0.379764 | 0.113795 | 1.267374 |

IVW, Inverse variance weighted

**Supplementary Table 7. Two-Sample MR Estimates of Relationship Between Plasma cortisol and Epilepsy**

| **Exposure: Plasma cortisol Outcome: Epilepsy** | | | | | | | |
| --- | --- | --- | --- | --- | --- | --- | --- |
| **Method** | **Beta** | **SE** | **P**-**value** | **or** | **or_lci95** | **or_uci95** |  |
| MR Egger | -0.623038 | 2.713106 | 0.856298 | 0.536313 | 0.002630 | 109.360428 |  |
| Maximum likelihood | 0.696654 | 0.347087 | 0.044734 | 2.007025 | 1.016497 | 3.962774 |  |
| WM* | 0.736606 | 0.405526 | 0.069306 | 2.088833 | 0.943437 | 4.624818 |  |
| IVW | 0.695206 | 0.341071 | 0.041520 | 2.004122 | 1.027066 | 3.910657 |  |

IVW, Inverse variance weighted; WM*, Weighted median

**Supplementary Table 8. Two-Sample MR Estimates of Relationship Between Plasma cortisol and Multiple Sclerosis**

| **Exposure: Plasma cortisol Outcome: Multiple Sclerosis** | | | | | | |
| --- | --- | --- | --- | --- | --- | --- |
| **Method** | **Beta** | **SE** | **P**-**value** | **or** | **or_lci95** | **or_uci95** |
| MR Egger | -0.647887 | 0.936129 | 0.614591 | 0.523150 | 0.083518 | 3.276991 |
| Maximum likelihood | -0.084521 | 0.118936 | 0.477305 | 0.918953 | 0.727869 | 1.160201 |
| WM* | -0.099681 | 0.142453 | 0.484083 | 0.905126 | 0.684622 | 1.196650 |
| IVW | -0.084030 | 0.118338 | 0.477651 | 0.919403 | 0.729079 | 1.159412 |

IVW, Inverse variance weighted; WM*, Weighted median

**Supplementary Table 9. Heterogeneity test Between Plasma cortisol and outcomes**

| **Method** | **Alzheimer’s disease** | **Vascular dementia** | **Parkinson’s disease with dementia** | **Dementia with Lewy bodies** | **Frontotemporal dementia** | **Epilepsy** | **Multiple Sclerosis** |
| --- | --- | --- | --- | --- | --- | --- | --- |
| **IVW** |  |  |  |  |  |  |  |
| Q | 1.62596005 | 1.707284643 | 0.253272211 | 0.40350206 | 0.691005688 | 0.317613149 | 0.728445502 |
| P-value | 0.443534351 | 0.425860981 | 0.881054225 | 0.817298386 | 0.405822519 | 0.853161366 | 0.694736424 |
| **MR Egger** |  |  |  |  |  |  |  |
| Q | 0.5134341 | 0.642311798 | 0.141542715 | 0.161266652 | / | 0.077742853 | 0.359755163 |
| P-value | 0.473656027 | 0.422874899 | 0.706752136 | 0.687993009 | / | 0.780379853 | 0.548642243 |

IVW, Inverse variance weighted

**Supplementary Table 10. Pleiotropy test Between Plasma cortisol and outcomes**

|  | **Alzheimer’s disease** | **Vascular dementia** | **Parkinson’s disease with dementia** | **Dementia with Lewy bodies** | **Epilepsy** | **Multiple Sclerosis** |
| --- | --- | --- | --- | --- | --- | --- |
| **egger intercept** | 0.003655315 | -0.251264971 | -0.144432223 | -0.090582618 | 0.114366942 | 0.04901433 |
| **SE** | 0.003465531 | 0.243479657 | 0.43209593 | 0.184045861 | 0.233513651 | 0.080722051 |
| **P**-**value** | 0.483036856 | 0.489982961 | 0.794636657 | 0.708830893 | 0.710066435 | 0.652599199 |

# Supplementary Figures


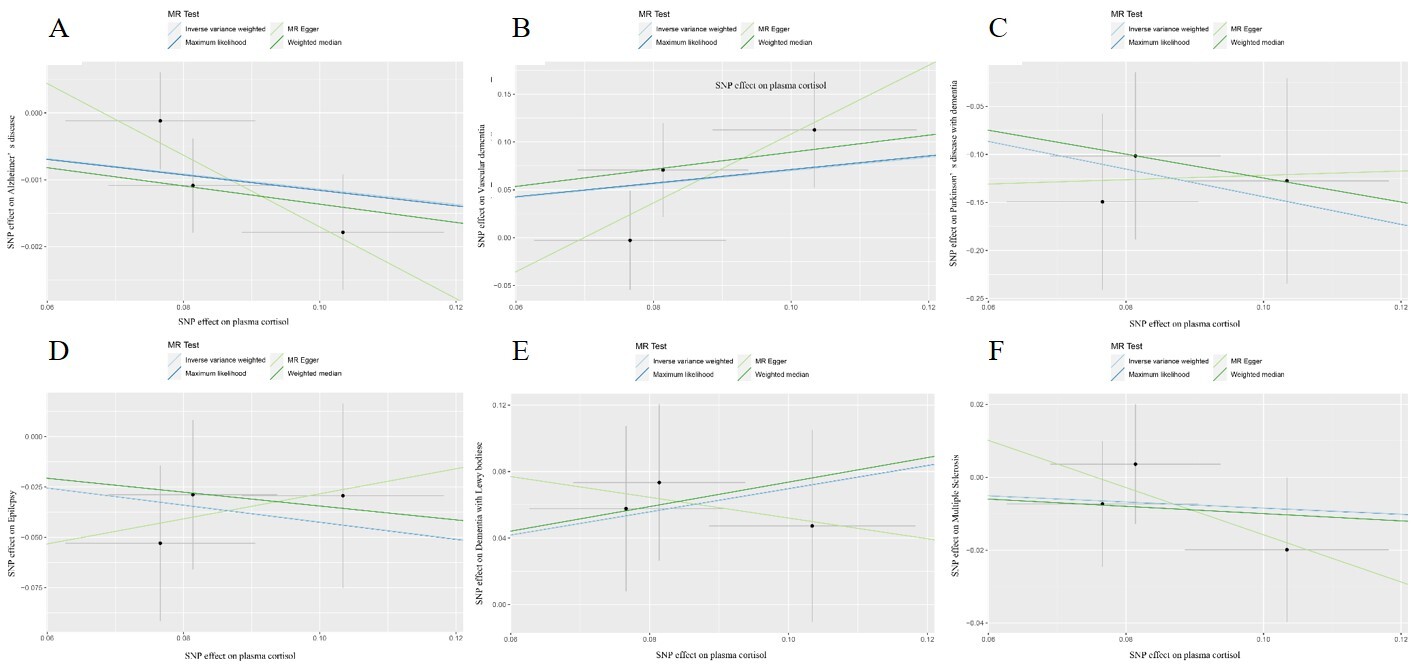


**Supplementary Figure 1. Scatter plots of the causal association between plasma cortisol and outcomes**

A: Alzheimer’s disease; B: Vascular dementia; C: Parkinson’s disease with dementia; D: Dementia with Lewy bodies; E: Epilepsy; F: Multiple Sclerosis


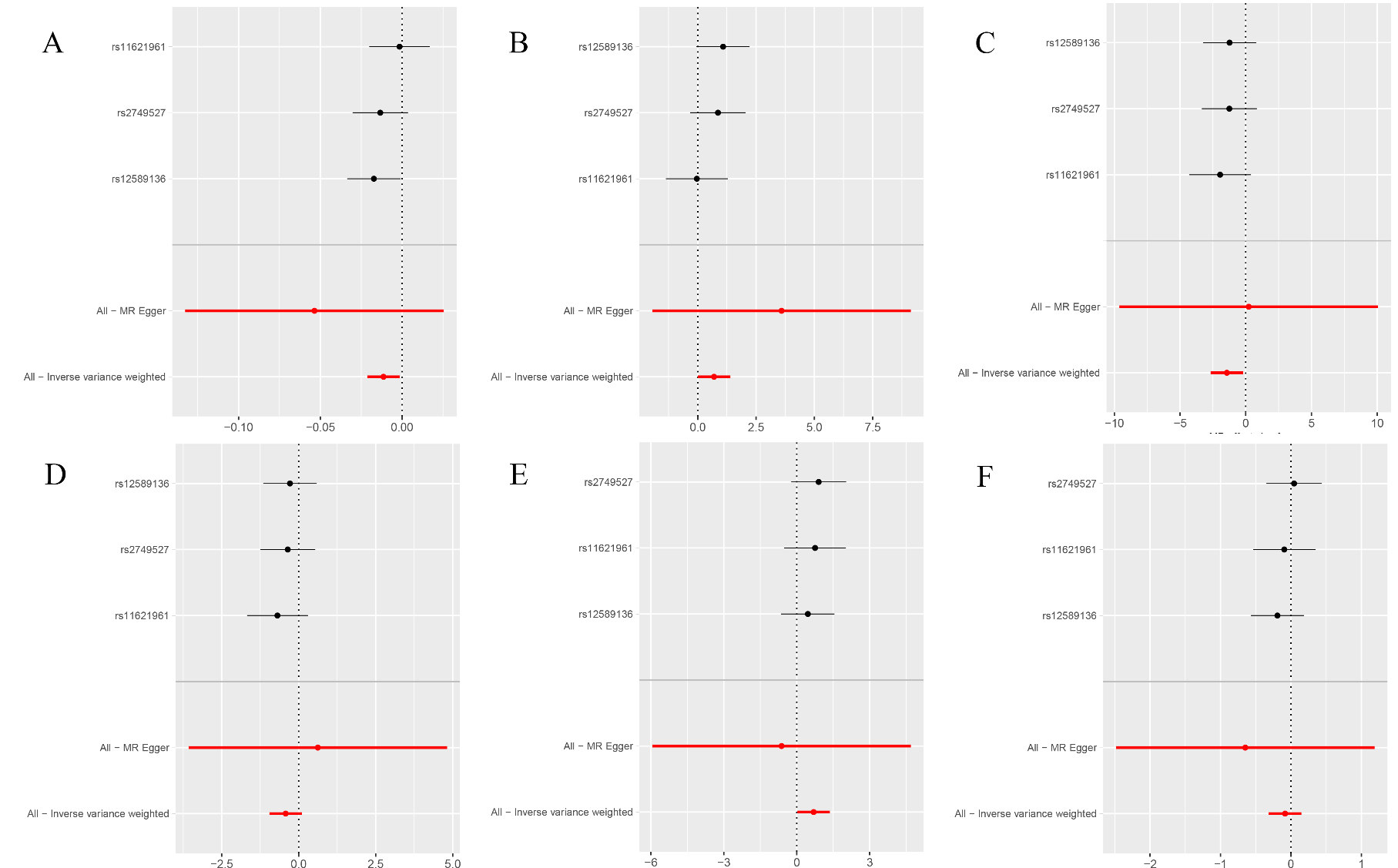


**Supplementary Figure 2. Forest plots of the causal association between plasma cortisol and outcomes**

A: Alzheimer’s disease; B: Vascular dementia; C: Parkinson’s disease with dementia; D: Dementia with Lewy bodies; E: Epilepsy; F: Multiple Sclerosis


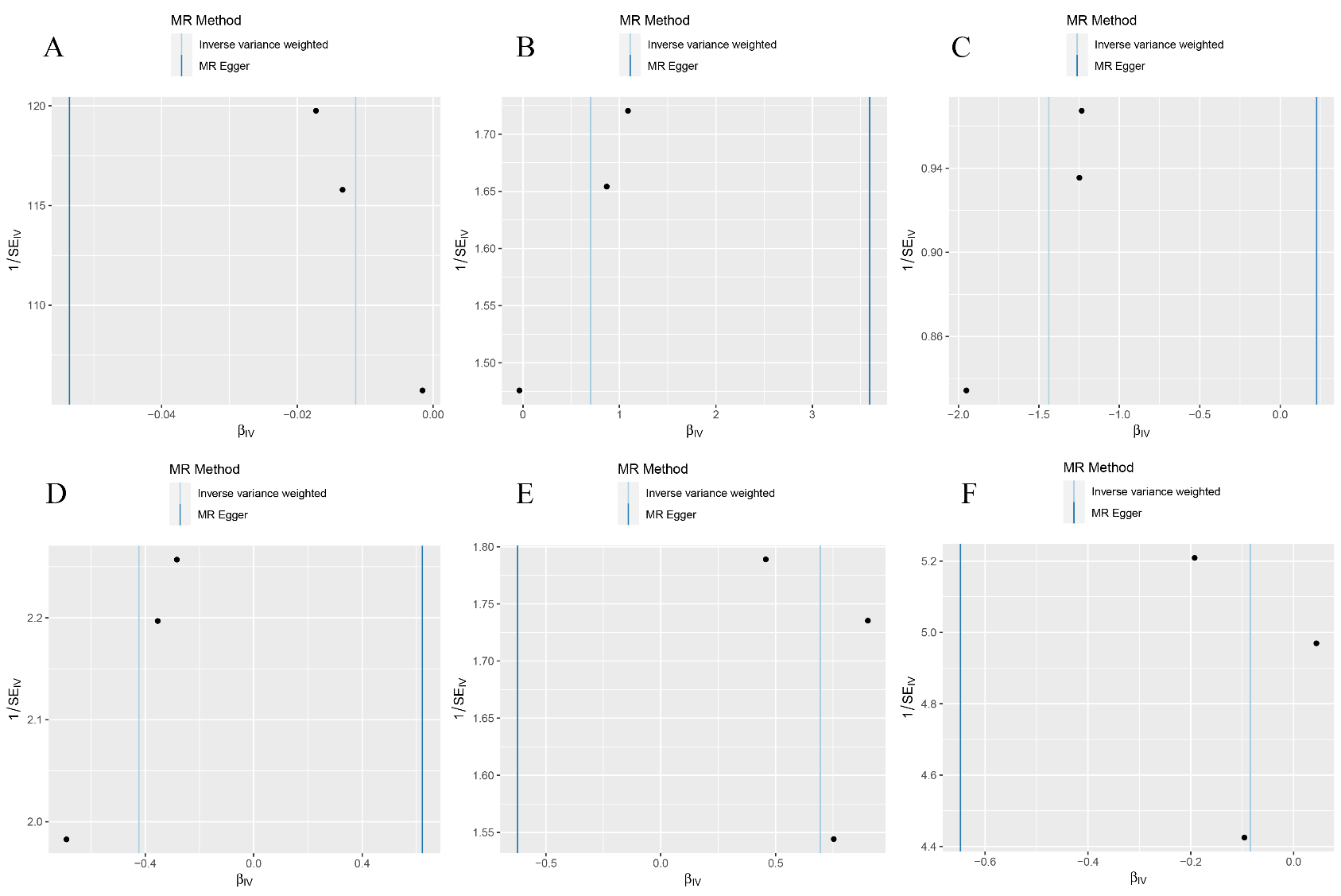


**Supplementary Figure 3. Funnel plots of the causal association between plasma cortisol and outcomes**

A: Alzheimer’s disease; B: Vascular dementia; C: Parkinson’s disease with dementia; D: Dementia with Lewy bodies; E: Epilepsy; F: Multiple Sclerosis


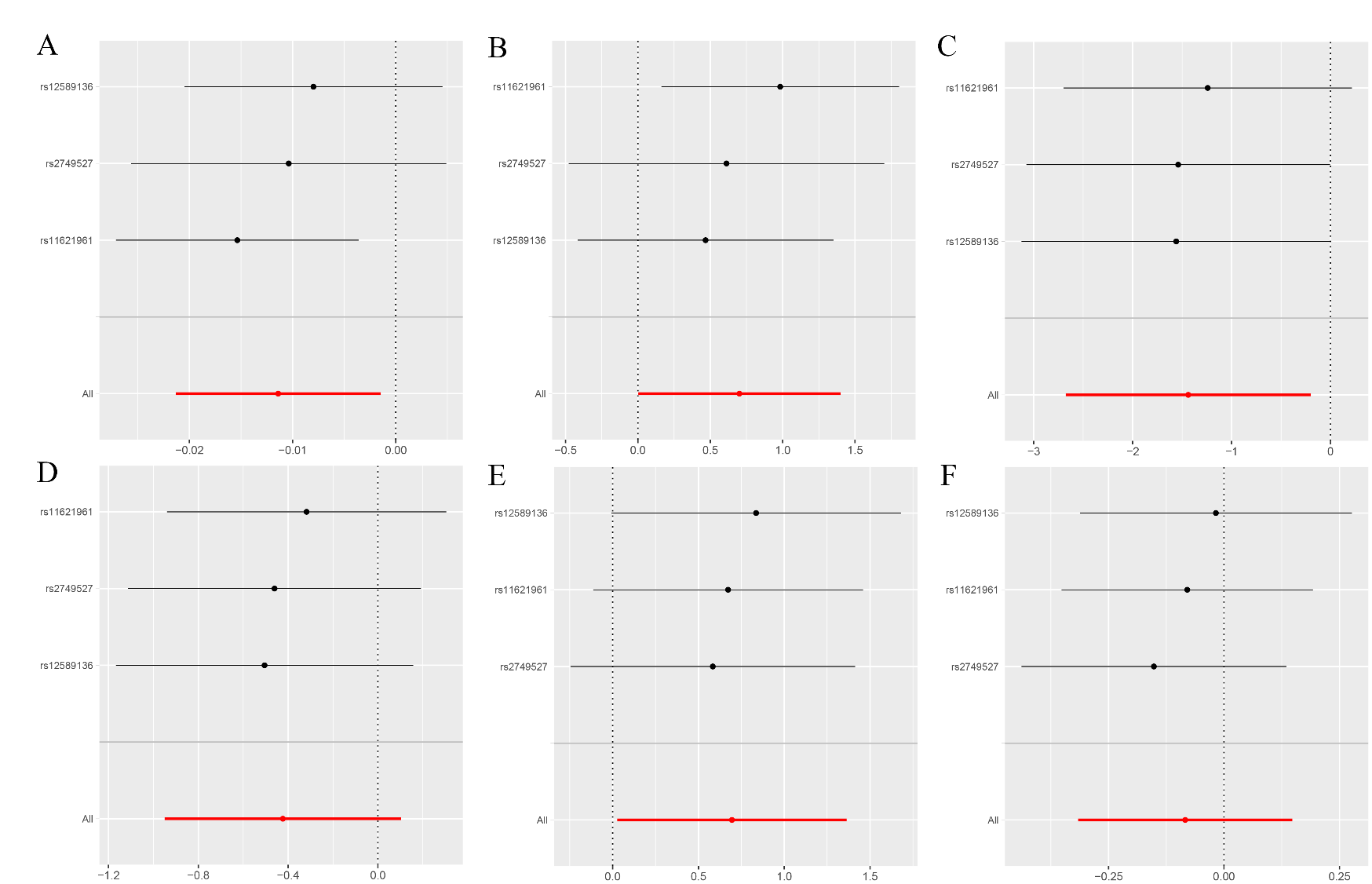


**Supplementary Figure 4. Leave-one-out method sensitivity analysis**

A: Alzheimer’s disease; B: Vascular dementia; C: Parkinson’s disease with dementia; D: Dementia with Lewy bodies; E: Epilepsy; F: Multiple Sclerosis
